# Management of temporary mechanical circulatory support devices in cath-lab and cardiac intensive care unit

**DOI:** 10.1093/ehjimp/qyad011

**Published:** 2023-07-28

**Authors:** Federico Fortuni, Filippo Zilio, Gianmarco Iannopollo, Giuseppe Ciliberti, Paolo Trambaiolo, Laura Ceriello, Francesca Musella, Pietro Scicchitano, Stefano Albani, Stefania Angela Di Fusco, Michele Massimo Gulizia, Domenico Gabrielli, Fabrizio Oliva, Furio Colivicchi

**Affiliations:** Department of Cardiology, San Giovanni Battista Hospital, via Massimo Arcamone, CAP 06034, Foligno (PG), Italy; Department of Cardiology, Leiden University Medical Center, Albinusdreef 2, 2330RC Leiden, The Netherlands; Department of Cardiology, Santa Chiara Hospital, Trento, Italy; Department of Cardiology, Maggiore Hospital Carlo Alberto Pizzardi, Bologna, Italy; Cardiology and Arrhythmology Clinic, Marche University Hospital, Ancona, Italy; Department of Cardiology, Sandro Pertini Hospital, Roma, Italy; Cardiology Department, Ospedale Civile G. Mazzini, Teramo, Italy; Division of Cardiology, Department of Medicine, Karolinska Institutet, Stockholm, Sweden; Cardiology Department, Santa Maria delle Grazie Hospital, Naples, Italy; Department of Cardiology, Hospital F. Perinei, Altamura, Italy; Division of Cardiology, U. Parini Hospital, Aosta, Italy; Department of Cardiology, Cardiovascular Institute Paris Sud, Massy, France; Clinical and Rehabilitation Cardiology Unit, San Filippo Neri Hospital, Rome, Italy; Cardiology Division, Garibaldi-Nesima Hospital, Catania, Italy; Cardio-Toraco-Vascular Department, San Camillo-Forlanini Hospital, Rome, Italy; Heart Care Foundation, Florence, Italy; Cardiologia 1, A. De Gasperis Cardicocenter, ASST Niguarda, Milan, Italy; Clinical and Rehabilitation Cardiology Unit, San Filippo Neri Hospital, Rome, Italy

**Keywords:** mechanical circulatory support, cardiogenic shock, hemodynamics, transthoracic echocardiography, transoesophageal echocardiography

## Abstract

Different temporary mechanical circulatory support (tMCS) devices are available and can be used to maintain end-organ perfusion while reducing cardiac work and myocardial oxygen demand. tMCS can provide support to the right ventricle, left ventricle, or both, and its use can be considered in emergency situations such as cardiogenic shock or in elective procedures such as high-risk percutaneous coronary intervention to prevent haemodynamic deterioration. Invasive and, most importantly, non-invasive haemodynamic parameters should be taken into account when choosing the type of tMCS device and its initiation and weaning timing, determining the need for a device upgrade, and screening for complications. In this context, ultrasound tools, specifically echocardiography, can provide important data. This review aims to provide a description of the different tMCS devices, the invasive and non-invasive tools and parameters to guide their management, and their advantages and drawbacks.

## Graphical Abstract

**Graphical Abstract:**
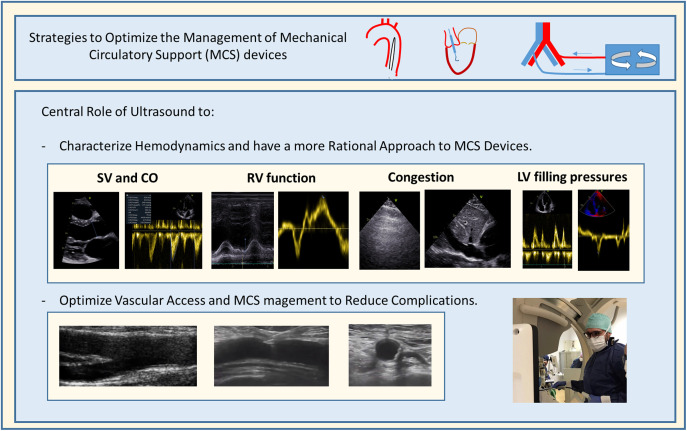
CO: cardiac output; LV: left ventricle; RV: right ventricle; SV: stroke volume.

## Introduction

Temporary mechanical circulatory support (tMCS) devices are useful to reduce cardiac stroke work and myocardial oxygen demand whilst maintaining end-organ perfusion,^1,2^ particularly in the setting of cardiogenic shock (CS) or high-risk percutaneous coronary intervention (HR-PCI). tMCS haemodynamic support can last from hours to weeks, and in the setting of shock may be used as a bridge to a decision, recovery, or long-term support or transplantation.^3^ Fully percutaneous or surgical configurations are available, and support can be directed to the right or left ventricle, or both.

Clinical and haemodynamic parameters should be considered to identify the presence of CS and choose the appropriate tMCS. While invasive haemodynamic data can be determinant in the management of CS patients,^4,5^ the use of pulmonary artery catheters (PAC) had been associated with an increased risk of in-hospital mortality, probably due to PAC-related complications.^6^ Therefore, non-invasive haemodynamic data obtained through echocardiography could be the key to optimize the treatment without incurring complications of invasive devices. In this setting, the most important advantages of echocardiography, beside non-invasiveness, are the wide availability and the relatively low costs. Nevertheless, measurement of the most interesting parameters [stroke volume (SV) index and the estimation of left ventricular (LV) filling pressures, that have been associated with risk of in-hospital mortality] may require high skills and suffer from significant limitations.^7–9^ Of note, ultrasound can also help to evaluate vascular accesses to reduce the complications linked to tMCS devices and this is of paramount importance in elective HR-PCI.

The aim of this review is to help clinicians dealing with patients supported by tMCS, or in need of these devices, to take full advantage of their potential benefits, exploiting the widely available ultrasound tools but also knowing the limitations and drawbacks.

Being this a review article, no new data were generated or analyzed in support of this research.

## Types of mechanical circulatory support devices

MCS devices can provide circulatory support in isolated LV failure, isolated right ventricular (RV) failure, biventricular failure, and CS.^1^

**Figure 1:**
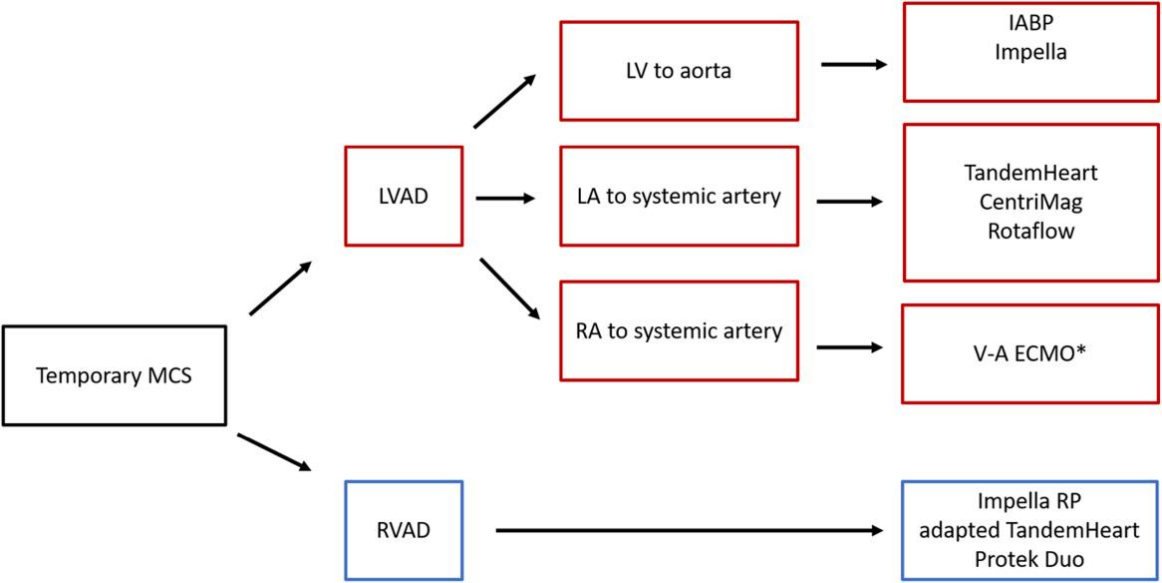
tMCS available according to the type of support and system configuration. IABP, intra-aortic balloon pump; LA, left atrium; LV, left ventricle; LVAD, left ventricular assist device; MCS, mechanical support circulatory support; RA, right atrium; RVAD, right ventricular assist device; V-A ECMO, veno-arterial extracorporeal membrane oxygenation. *V-A ECMO provides biventricular support.

MCS devices can be divided into the following categories: temporary or definitive, and with regard to the modality and position of implantation, para-corporeal, percutaneous, and intra-corporeal.^2^ Para-corporeal and percutaneous devices are considered tMCS, whereas intra-corporeal devices usually are for definitive use. This review will focus on temporary percutaneous devices (*Table 1* and *Figure 1*) most commonly used in intensive cardiac care units, thus of particular interest to cardiologists.

**Table 1:** Most common tMCS indications, contraindications, advantages, disadvantages, and complications

	Indications	Contraindications	Advantages	Disadvantages	Complications
**LV support devices**					
IABP	CS; HR-PCI	Severe AR, aortic aneurysm, aortic dissection, and peripheral vascular disease	Ease of insertion (bedside insertion); no or minimal anticoagulation needed.	Minimal haemodynamic support, poor LV unloading	Stroke, limb ischaemia, thrombocytopenia, infection
Impella	CS; HR-PCI	LV mural thrombus, mechanical aortic valve or heart constrictive device, very severe aortic stenosis, moderate to severe aortic insufficiency, severe peripheral artery disease	Direct ventricular unloading	Anticoagulation with heparin is mandatory	Haemolysis, bleeding, vascular injury, infection, and pump migration
TandemHeart	CS; severe LV disfunction; MI acute mechanical complications	AR and peripheral vascular disease	LV volume unloading; possible respiratory support with extracorporeal oxygenator in series with TandemHeart system; can be used in the presence of LV thrombus	Immobilization of the patient is required	Bleeding, thromboembolism, and limb ischaemia; cardiac wall perforation, aortic root puncture, pericardial effusion, or tamponade
**RV support devices**					
Impella RP	RV failure after cardiac surgery; combined use with LVAD	Similar to other Impella devices; significant tricuspid and pulmonary regurgitation.	RV unloading; an oxygenator can be spliced into the circuit (oxy-RVAD configuration).	Anticoagulation with heparin is mandatory	Haemolysis, bleeding, vascular injury, infection, and pump migration
Adapted TandemHeart/Protek Duo	RV failure following LVAD implantation; pulmonary hypertensive crisis	Limited data	An oxygenator can be spliced into the circuit (oxy-RVAD configuration).	Limited data	Limited data
**Biventricular support device**					
V-A ECMO	CS; advanced heart failure	Severe irreversible non-cardiac organ failure limiting survival; irreversible cardiac failure if transplantation or long-term VAD will not be considered; severe aortic insufficiency; aortic dissection.	Addition of respiratory support	Does not allow complete LV unloading	Multiorgan failure, prolonged cardiopulmonary resuscitation, aortic dissection, and severe AR

ECMO, extracorporeal Membrane Oxygenation; CS, cardiogenic shock; HR-PCI, high-risk percutaneous coronary intervention; IABP, intra-aortic balloon pump; LV, left ventricle; LVAD, Left Ventricular Assist Device; MCS, mechanical circulatory support; MI, myocardial infarction; PCI, percutaneous coronary intervention; RV, right ventricle; RVAD, right ventricle assist device.

For LV support, available devices include intra-aortic balloon pump (IABP), Impella, TandemHeart, iVAC 2L, CentriMag, and Rotaflow; for RV support, Impella RP, and TandemHeart RV/Protek Duo; for biventricular support, veno-arterial extracorporeal membrane oxygenation (VA-ECMO). *Table 1* and *Figure 1* summarize the main characteristics of each tMCS.

## Invasive and non-invasive hemodynamic monitoring systems in patients with cardiogenic shock

Several invasive and non-invasive monitoring systems and devices are available to evaluate the haemodynamic status, identify the presence of CS and evaluate the efficacy of CS treatment. Physical examination may be helpful and represents the oldest method to evaluate CS patients. The 2019 SCAI (Society for Cardiovascular Angiography and Interventions) Clinical Expert Consensus Statement on Classification of CS was developed based on physical examination in conjunction with biochemical and invasive haemodynamic data^10^ (*Table 2*). Signs and symptoms of systemic and pulmonary congestion and hypoperfusion are considered to classify the worsening SCAI stages of CS.^10^ Although physical exam plays a pivotal role in the initial identification and classification of CS, has been shown to have low accuracy^11^ and is less adequate to identify the cause of shock and for risk stratification^12^ than invasive parameters.

**Table 2:** SCAI (Society for Cardiovascular Angiography and Interventions) shock stages

SCAI shock stages	
A (at risk)	A patient who is not currently experiencing signs or symptoms of CS, but is at risk for its development. These patients may include those with large acute MI or prior infarction acute and/or acute on chronic heart failure symptoms.
B (beginning)	A patient who has clinical evidence of relative hypotension or tachycardia without hypoperfusion.
C (classic)	A patient that manifests with hypoperfusion that requires intervention (inotrope, vasopressor, or mechanical support, including ECMO) beyond volume resuscitation to restore perfusion. These patients typically present with relative hypotension.
D (deteriorating)	A patient that is similar to category C but is getting worse. They have failure to respond to initial interventions.
E (extremis)	A patient that is experiencing cardiac arrest with ongoing CPR and/or ECMO, is supported by multiple interventions.

CPR, cardiopulmonary resuscitation; CS, cardiogenic shock; ECMO, extracorporeal membrane oxygenation.

PAC is the mainstay of invasive haemodynamic monitoring. Although their value in the past has been questioned due to potential complications,^13^ more recent studies have shown that they may improve prognosis.^5^ PAC provides direct measurements of intra-cardiac and vascular pressures as well as indices of cardiac output (CO) and cardiac index (CI). CO can be measured by two techniques in clinical practice, indirect Fick, and bolus thermodilution. Each technique has its pitfalls related to the presence of significant tricuspid regurgitation, atrial fibrillation, and low-output conditions; nevertheless, thermodilution showed higher prognostic value^14^ and should be preferred over the indirect Fick method in clinical practice.

Several minimally invasive monitoring systems requiring central venous access are also available. One example is the pulse contour CO (PiCCO) monitoring system. PiCCO is performed by injecting a cold fluid bolus via a central venous catheter and measuring the resultant thermodilution via a thermistor-tipped femoral artery catheter and has shown good agreement with PAC-derived measurements.^15^ After being calibrated with thermodilution, PiCCO can use pulse contour analysis for continuous CO and SV measurements. Systems that estimate SV and CO based only on pulse contour analysis derived from an arterial line are also available, but they have been deeply criticized for their low accuracy compared with PAC measurements.^16^

**Figure 2:**
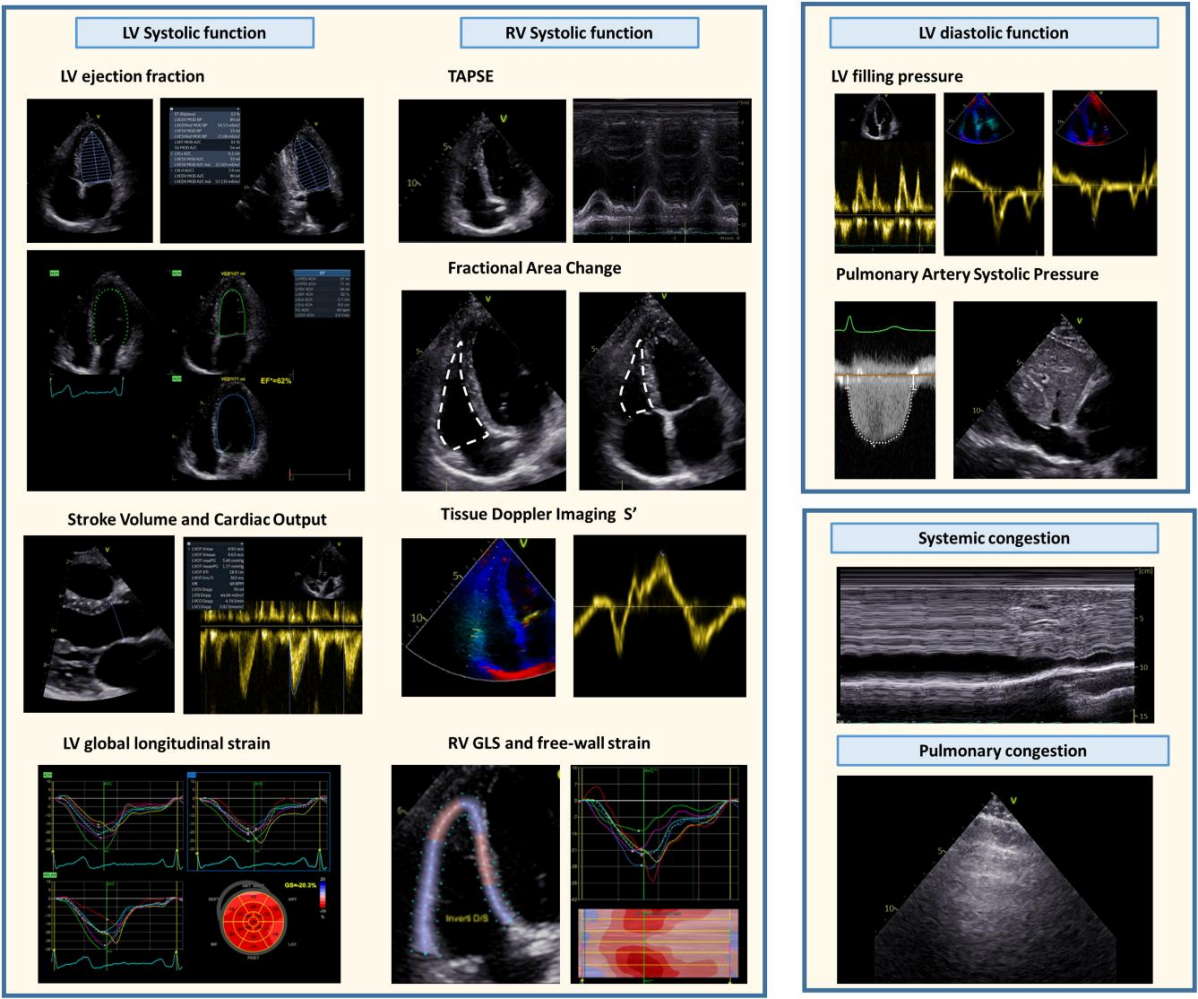
Most common conventional and advanced echocardiography-derived haemodynamic parameters. GLS: global longitudinal strain; LV: left ventricular; RV: right ventricular; TAPSE: tricuspid annular plane systolic excursion.

Finally, transthoracic and transoesophageal echocardiography (TTE and TOE) are very useful in patients with CS for several reasons. First, they can help in determining the cause of CS. This can be very important in emergency conditions such as CS associated with critical aortic or mitral stenosis or regurgitation, hemodynamically significant intra-cardiac shunts as well as aortic dissection and RV failure due to massive pulmonary embolism, TOE and TTE may be determinant to have a prompt diagnosis and guide either their percutaneous or surgical treatment. Moreover, they offer the opportunity of measuring several haemodynamic parameters (*Table 3* and *Figure 2*) that can be compared over time to evaluate the need of using tMCS systems, choose which one is needed, help in placing and managing them, and decide when potentiation is needed or weaning can be started. SV and CO can be derived from 2D and 3D TTE based on left ventricular outflow tract (LVOT) dimensions and pulsed wave Doppler measurements at the LVOT level and showed to be consistent with invasive measurements.^17^ Also less advanced echocardiographic parameters of LV systolic function such as 2D LV ejection fraction (LVEF) had recently shown a significant correlation with SCAI stages and an independent association with mortality in patients hospitalized due to acute heart failure.^9^ Recent studies reopened the discussion about the need for invasive haemodynamic monitoring due to the fact that echocardiographic-derived haemodynamic measurements not only provide the above-mentioned benefits but have also demonstrated an association with shock stages.^8^ Not only TTE and TOE but also vascular and lung ultrasound are useful and should be used in patients with CS to assess end-organ perfusion and congestion.^18^ Renal and splenic Doppler resistive indices as well as transcranial Doppler ultrasound may be used to assess renal, splenic, and cerebral perfusion, respectively. Systemic congestion can be assessed by the evaluation of inferior vena cava diameter and collapsibility but also by analyzing the intra-renal, hepatic, and portal venous flow with Doppler ultrasound.^18^ Conversely, lung ultrasound and the presence and quantity of B-lines can be used as a measure of pulmonary congestion.^18^

**Table 3:** Most common echocardiographic-derived haemodynamic parameters

Echocardiographic parameter	Measures needed	Limitations	How to	Normal values
Stroke volume (SV)	LVOT diameter Pulsed-wave Doppler at the LVOT level	LVOT shape which is not circular Low accuracy if significant AR is present	LVOT VTI × LVOT area SV index can be derived by dividing SV per BSA	50–100 mL per beat
Cardiac output (CO)	LVOT diameter Pulsed-wave Doppler at the LVOT level HR	LVOT shape which is not circular Low accuracy if significant AR is present Irregular HR	(LVOT VTI × LVOT area)×HR CI can be derived dividing CO per BSA	5–6 L/min
CPO	CO (L/min) (see above) Mean arterial pressure (MAP)	Limited data for high RAP relative to MAP [79]	(COxMAP)/451	Approximately 1 W
RAP	IVC diameter IVC respiratory variation	Mechanical ventilation Significant TR	Non-dilated IVC with > 50% inspiratory collapse → 3–5 mmHg Dilated IVC with > 50% inspiratory collapse → 8–10 mmHg Non-dilated IVC with < 50% inspiratory collapse → 8–10 mmHg Dilated IVC with < 50% inspiratory collapse → 13–15 mmHg	3–5 mmHg
Pulmonary artery systolic pressure (PAPS)	Maximum TR velocity (Vmax) derived with continuous wave Doppler IVC diameter and collapsibility	Mechanical ventilation Significant TR RV dysfunction	(4×Vmax^2^) + RAP	<35 mmHg
LV filling pressure [Medial early mitral valve inflow velocity to early diastolic annular velocity ratio (E/e’ ratio)]	Early mitral inflow velocity derived with pulsed wave Doppler at the level of the mitral valve (E) Mitral annular early diastolic velocity derived with tissue Doppler imaging (e’)	Significant mitral regurgitation Mechanical ventilation Pericardial effusion Constrictive pericarditis	E/e’	<14
B lines	Lung ultrasound scans	Interstitial lung disease Subcutaneous emphysema High acoustic impedance Large thoracic dressings	The number of B-lines and the number of thoracic segments involved correlate with the level of pulmonary congestion	None

AR, aortic regurgitation; BSA, body surface area; HR, heart rate; IVC, inferior vena cava; LV, left ventricular; LVOT, left ventricular outflow tract; RV, right ventricle; RAP, right atrial pressure; SV, stroke volume; TR, tricuspid regurgitation; VTI, velocity time integral.

**Figure 3:**
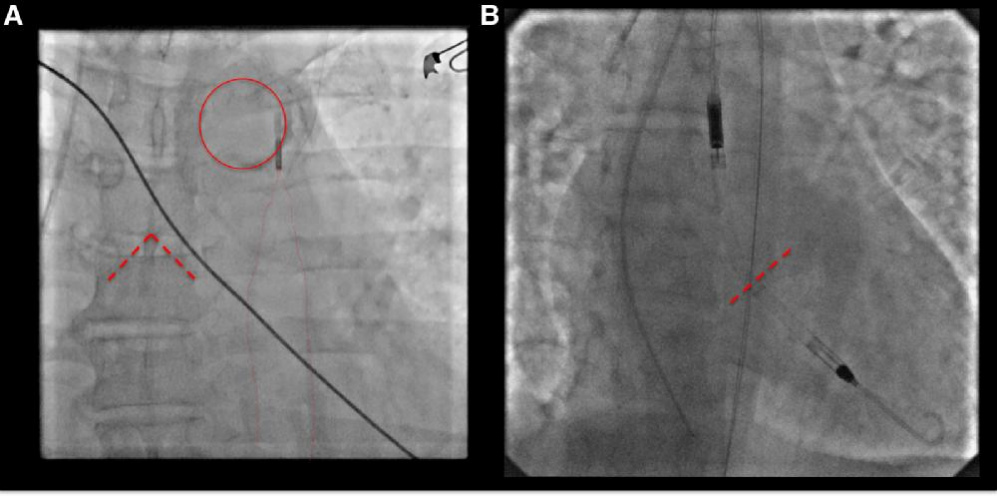
Fluoroscopy imaging to gain optimal peripheral MCS device positioning. Panel A shows the fluoroscopic image after IABP placement. The circle indicates the aortic knob which marks the beginning of the thoracic descending aorta where the proximal IABP marker should be placed (2nd–3rd intercostal space). The dashed line illustrates the tracheal carina while the dotted line shows the inflated IABP within the descendent thoracic aorta. Panel B shows the fluoroscopic image after Impella placement. The dotted line indicates the aortic valve plane where the dedicated device marker should be located. IABP: intra-aortic balloon pump; MCS: mechanical circulatory support.

TTE and TOE can help detect possible conditions that can contraindicate the use of tMCS (*Table 1*), for instance, the presence of aortic dissection, which needs prompt surgical intervention. Echocardiography can also help identify other contraindication to tMCS (*Table 1*), such as severe aortic regurgitation (AR) that contraindicates the use of IABP. Concerning IABP, TTE, and/or TOE can also be used to check the position of the distal tip of the device, which should be located 2–3 cm distal to the left subclavian artery in the descending aorta (*Figure 3*).

TTE and TOE are also very important to obtain and eventually regain an adequate positioning of peripheral ventricular assist devices (pVAD), such as Impella. A systematic imaging approach (*Table 4*) in the different stages of pVAD implantation should be used and may be useful to prevent, detect and manage complications. Adequate device positioning is pivotal for optimal circulatory support^19^ and should be firstly checked while placing it with fluoroscopy in the cath-lab (*Figure 3*) and then verified with TOE and/or TTE. The inlet opening of a left-sided Impella device should be located at the mid-ventricular level, which is usually 3.5–4 cm below the aortic valve. Concerning right-sided pVAD, the outflow opening should be located 2–4 cm into the pulmonary artery. A too-deep location in the ventricle may result in haemolysis, suction deficits, and ventricular arrhythmias; conversely, a too-distal location and the position of the inlet into the vascular system can cause inefficient circulatory support.^19^ In most cases, the correct position of a left-sided Impella can be checked with TTE using the parasternal long-axis view or the apical three- or five-chamber view, checking the distance between the ventricular portion of the device and the aortic valve, which should be around 3.5 cm without including the pigtail in this calculation.^19^ When the acoustic window is not adequate, TOE may be necessary and, in these cases, the most useful view is the long axis view imaging the LVOT and aortic valve. In case of device displacement, TTE or TOE can guide device re-positioning. Chest X-ray and fluoroscopy may be useful in some cases to check the position of the device (*Figure 3*), especially in right-sided pVAD, where the distal position on the pulmonary trunk should be verified.

**Table 4:** Step-by-step echocardiographic approach for the management of peripheral left VAD

Phase	Parameters to assess
Pre-implantation	Screen for relative/absolute contraindications: Atrial septal defect can create a right-to-left shunt. Severe AR that can worsen LV output. RV systolic dysfunction. Aortic dissection or complicated plaque. Intra-cardiac thrombi. AML and inter-ventricular septum assessment to avoid LVOT obstruction.
Post-implantation	Assess correct device positioning: Check the distance between the ventricular portion of the device and the aortic annulus. Verify the presence of the mosaic flow pattern above the aortic valve and sinus of Valsalva with colour Doppler imaging. Verify the spatial relationship with the inter-ventricular septum and AML to avoid LVOT obstruction.
Device re-positioning	Real-time TOE or TTE assessment to monitor and re-gain the adequate position.
Screen for complications	Assess patient volemia. RV failure screening. Detect cardiac tamponade

AML, anterior mitral leaflet; LV, left ventricular; LVOT, left ventricular outflow tract; RV, right ventricular; TTE, transthoracic echocardiography; TOE, transoesophageal echocardiography; VAD, ventricular assist device.

TTE and possibly TOE also have a pivotal role in VA-ECMO.^20^ In the initial pre-VA-ECMO echocardiographic assessment, in addition to the characterization of CS and the decision to start and when to start VA-ECMO, they can assess potential contraindications such as aortic dissection. Moreover, the presence of complicated atherosclerotic plaques in the thoracic aorta identified with TOE may prompt the need for surgical over percutaneous cannulation. VA-ECMO increases LV afterload and therefore can lead to a worsening of left-sided valvular regurgitation, which should be carefully assessed before and after VA-ECMO initiation.^21^ TOE plays a fundamental role in guiding and confirming the right position of the VA-ECMO cannulas. The bicaval view should be used to confirm the position of the venous cannula in the proximal part of the inferior vena cava or right atrium, whereas according to the different arterial access used (peripheral cannulation of the axillary or femoral artery or direct central surgical cannulation of the ascending aorta) TOE or general vascular ultrasound may be used to confirm the arterial cannula position.^20^

## Timing of initiation of mechanical support devices

### Cardiogenic shock

CS is mainly caused (80% of the cases) by LV dysfunction secondary to acute myocardial infarction (MI) and is associated with high mortality.^22^ The literature underpinning the use of tMCS in CS is controversial, however, their use is recommended by international guidelines, mainly supported by expert opinions.^1,2,7,23,24^ tMCS primarily aims to restore CO in patients with CS or in case of refractory cardiac arrest.

**Figure 4:**
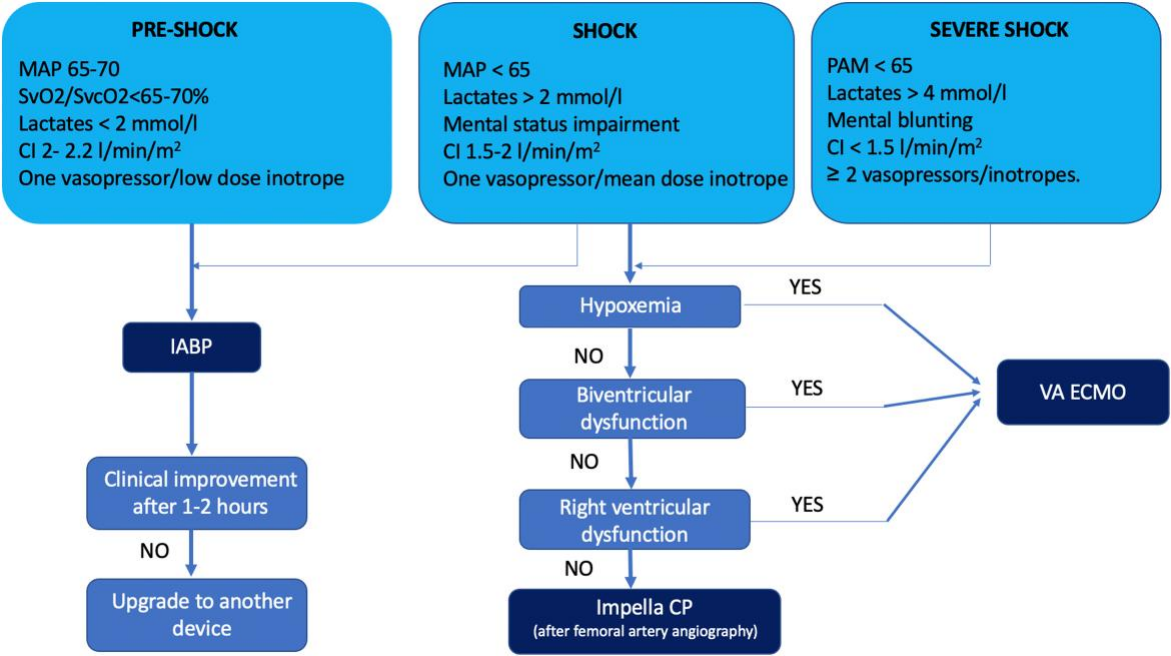
Flowchart on the management of MCS devices in patients with CS. CI, cardiac index; IABP, intra-aortic balloon pump; MAP mean arterial pressure; VA-ECMO, Veno arterial ExtraCorporeal Membrane Oxygenation. Adapted from Battistoni et al.^25^

A multi-parametric evaluation (clinical, laboratory, and haemodynamic parameters) becomes, therefore, essential to block the shock cascade (*Tables 2* and *3*, *Figure 4*) and also to predict patient prognosis and eventual response to treatment. Recently a three-axis model to evaluate CS patients had been proposed and this integrates three domains: (i) shock severity, (ii) clinical phenotype, and (iii) risk modifiers as distinct constructs that must be considered during clinical decision making.^26^ When evaluating CS patients on the potential need for tMCS, the high mortality, costs, and ethical issues related to CS should be taken into account to prevent futility and several multi-parametric scores (such as the APACHE III) may be of help.^27^ From a pragmatic point of view, an initial evaluation could be represented by invasive arterial pressure monitoring (systolic and mean arterial pressure [MAP], pressure waveform, blood gas analysis to evaluate PaO_2_/FiO_2_, and lactates), central venous access (central venous pressure and SvO_2_/SvCO_2_ analysis), and urinary catheter (to evaluate diuresis). A second-level multi-parametric approach could be represented by haemodynamic monitoring with a pulse contour-based system or PAC to evaluate CO and pulmonary capillary wedge pressure.^28^ Moreover, additional haemodynamic parameters could be derived from standard invasive parameters, such as cardiac power output (CPO) [CO (L/min) multiplied by MAP and divided by 451] and pulmonary artery pulsatility index (PAPi) (the ratio of pulmonary artery pulse pressure to right atrial pressure) that are associated with in-hospital mortality in CS patients.^29^ In this setting, TTE and TOE are essential to differentiate between different shock conditions and specifically to identify the aetiology of CS, evaluate patient volemic status in association with lung ultrasound (lung congestion), describe patient hemodynamics (*Table 3*), guide the choice of therapies, assess response to interventions and evaluate the adequacy and monitoring of tMCS.^19,30^ A recent study showed that SCAI shock stages were closely related to standard echocardiographic parameters such as LVEF and that haemodynamic parameters derived from echocardiography were associated with worsening shock stages and in-hospital mortality.^9^ Moreover, patients who had a non-invasive haemodynamic assessment with echocardiography at admission had a lower length of hospital stay, lower use of inotropes, and most importantly, were more likely to survive hospitalization.^8,9^ This data may underline the beneficial effect of a more rational and pathophysiology-based approach to CS with the help of echocardiography, which could also have a strong impact on patient management and prognosis.

The failure to achieve pre-established targets (lactates < 2 mmol/L; SvO_2_/SvcO_2_ > 65–70%, MAP > 65 mmHg, heart rate < 110 bpm) despite adequate volume filling and pH correction, indicates the need for higher haemodynamic support apart from pharmacological options.^25^ It could be therefore considered the early use of tMCS, choosing the most appropriate based upon concomitant clinical conditions (e.g. RV dysfunction, hypoxia, and severity of CS) and vascular access viability. Potential benefit from Impella placement before PCI in acute MI complicated by CS is supported by several studies.^31^ Nevertheless, in a recent propensity-matched analysis of patients undergoing PCI for acute MI complicated by CS, Impella use was associated with increased short-term and 1-year risk of mortality, bleeding, and cost compared with IABP.^32^ These controversial data will be reconsidered after the results of the ongoing DanGer Trial, which will test the hypothesis that tMCS with Impella CP improves survival in patients with ST-elevation myocardial infarction complicated by CS compared to conventional treatment.^33^ In waiting for the results of this study, the recently published ECMO-CS trial did not show a benefit of early support with VA-ECMO vs. a conservative approach in SCAI shock stages D-E patients on the study primary endpoint (i.e. death from any cause, resuscitated circulatory arrest, and use of another MCS device at 30 days), nor for the individual components of the composite primary endpoint.^34^ Until randomized data will be available, decisions regarding the timing of tMCS initiation should be based on individual-patient risk/benefit assessment, taking into account shock severity and comorbidities.^7^ To this purpose, considered the minimal risk and the potential great benefits both in guiding management and on prognosis, all the data obtained from non-invasive (mainly echocardiography) or minimally invasive tools should be considered to aid a tailored approach for each patient.

### High-risk PCI

The term HR-PCI does not have a unique definition and refers to a spectrum of procedures with one or more of the following features: unprotected left main coronary artery disease, intervention of the last patent vessel, LVEF < 35%, complex three-vessel disease, or comorbidities including severe aortic stenosis or mitral regurgitation.^35^ Protected PCI is the concept of treating high-risk patients with the preventive placement of tMCS that may reduce haemodynamic intolerance risk.

Technical issues and procedural planning need to be adequately evaluated when dealing with patients with low LVEF due to the impaired haemodynamic stability arising from coronary flow blockage during balloon inflations in complex PCI or from PCI complications.^22^ There is no definite threshold to indicate the need for any tMCS, however, reduced LVEF increases the likelihood of haemodynamic instability during complex PCI procedures.^36^ Invasive LV end-diastolic pressure demonstrated to impact prognosis in invasively managed acute coronary syndromes.^37^ Therefore, evaluation of diastolic function with echocardiography (*Table 3* and *Figure 2*) could be considered in risk stratification alongside LV systolic function. In this scenario, tMCS might reduce LV filling pressures and prevent haemodynamic collapse during multiple revascularizations. Multivessel PCI with the Impella placement showed better support than IABP.^38^ Nevertheless, this data will be further investigated in the ongoing PROTECT IV randomized trial.^39^

## Vascular access management

Complications of tMCS are common, remain high despite technological progress, and are associated with increased mortality and costs.^40^ Lemor et al. reported vascular complications ranging from 3% to 15%. Moreover, the mortality of patients with vascular complications was significantly higher when compared with those without.^41^ Prior studies have reported vascular complications of around 4% for IABP, 6–8% for Impella, and 12–15% for ECMO.^31,42,43^

Previous trials have shown the potential benefits of ultrasound guidance and vascular closure devices for femoral artery puncture as compared to traditional approaches. The FAUST trial showed that ultrasound guidance reduces the number of attempts, time to access, risk of venipunctures, and vascular complications in femoral arterial access.^43^ Similarly, the PETRONIO registry showed that the application and the expertise acquired in ultrasound-guided puncture and suture-like hemostasis reduces in-hospital access site bleeding and vascular complications in an unselected population undergoing different types of procedures.^44^

**Figure 5:**
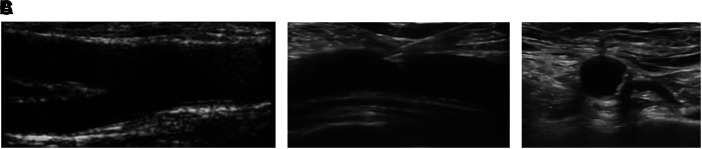
Ultrasound-guided femoral puncture. (*A*) Obtain a longitudinal view of the femoral bifurcation; (*B*) Puncture of the CFA on top of the femoral head with a clear tenting; (*C*) Keep tenting and rotate anti-clockwise the probe and enter the vessel on the anterior wall.

Ultrasound-guided technique consists of imaging the femoral artery bifurcation in the short and long axis plane until the common femoral artery (CFA) is visualized (*Figure 5*). A reasonable calcium-free spot should be identified before puncturing CFA. Local anaesthetic is administered subcutaneously under direct visualization with ultrasound. The needle is advanced through the anterior wall of the vessel considered as a puncture reference in the short axis plane. Longitudinal view could be performed to evaluate the correct height and freedom of calcification. The guide wire could be advanced through the needle into the femoral artery when adequate pulsatile blood flow is visualized. Further evaluation with ultrasound could be considered before sheath placement. It is recommended to perform a femoral artery angiogram after sheath placement to evaluate possible complications before administration of intra-arterial heparin.

The minimal lumen diameters of the CFA access site accommodating a tMCS device depend on the sheath size and the cannula diameter; specifically, it should be >4.5 mm for Impella, > 2 mm for IABP, and from 5 to 7.5 mm for VA-ECMO (*Table 5*). Peripheral vascular disease represents a risk factor for bleeding and vascular complications after tMCS placement.^41^ Procedural planning is, therefore, essential to avoid vascular events and to streamline the procedure. In the scenario of a CS, the fast evaluation with ultrasound of CFA can guide in choosing the most appropriate tMCS device (*Table 5*). In the setting of HR-PCI, procedural planning with invasive angiography or 3D angiography multislice computed tomography scan can be of help to evaluate the feasibility of percutaneous transfemoral access or consider alternative strategies (open surgical femoral access or transaxillary access).

**Table 5:** Access site characteristics of different mechanical cardiac support devices

	MLD femoral artery	Sheat/Cannula size	Hemostasis
IABP	>2 mm	7–8.5 Fr	1 Proglide
Impella CP	>4.5 mm	14 Fr	2 Proglide, or 1 Manta system, or 1 Proglide and 1 Angioseal
VA-ECMO	>5–7.5 mm (depending on the cannula size)	8–23 Fr (depending on patient weight)	Up to 3 Proglide.

IABP, intra-aortic balloon pump; MLD, minimal lumen diameter; VA-ECMO, veno-arterial extracorporeal membrane oxygenation.

In case of haemodynamic need for Impella support and a concomitant severe peripheral vasculopathy, new alternative strategies adopted from transcatheter aortic valve implantation experience could be considered. A valid option in elective procedures (e.g. HR-PCI) is the insertion of Impella from the transaxillary access.^45^ Alternatively, the use of intravascular lithotripsy or balloon angioplasty of the iliac-femoral artery axis to facilitate Impella transfemoral access had already been described in the literature.^46^ Furthermore, transcaval access had also been adopted in emergency cases to advance the Impella device.^47^

## Screening for complications

CS patients treated with a tMCS device can experience several complications, that can be related to preexisting comorbidities, to the evolution of the haemodynamic deterioration, or malfunctioning or complications related to the tMCS and associated treatments. The main complications related to any tMCS devices are bleeding events, with a mean incidence of 30%.^48^ Those events are mainly due to the anticoagulant treatment needed to prevent pump thrombosis.^49^ Another possible complication of tMCS is haemolysis, whose incidence ranges from 8% to 63%.^19^ Haemolysis can be treated by optimizing the volume status, re-positioning the device, or intensifying anticoagulant treatment (in case of pump thrombosis). The Impella console may be very helpful to screen for device malfunction. The console provides the following data: (i) continuous pressure measurement at the outflow of the catheter and (ii) electric current of the motor pump of the device which should be pulsatile because the device works based on the pressure difference between the inlet and outlet cannulas which should differ between systole and diastole. If the Impella dislocates and the inlet and outlet cannulas are in the same compartment, the electric current of the motor pump will flatten and the position of the device should be checked with TTE or TOE.

The presence of organ hypoperfusion can be detected based on clinical, laboratory, ultrasound, and haemodynamic data and may indicate the need for a device upgrade (*Figure 4*). Nevertheless, hypoperfusion may frequently be the consequence of increased tissue oxygen demand, systemic inflammatory response syndrome, and/or device malfunctioning. Those conditions should be identified and treated before considering a device upgrade.^19^ TTE can be helpful in identifying some frequent and treatable complications such as RV failure in patients with left-sided pVAD and cardiac tamponade, which may have an atypical presentation in patients with tMCS.^19^ In patients with left-sided pVAD, the RV is responsible for the transmission of the systemic preload to the LV. When the RV cannot pump enough blood to the left side, the central venous pressure increases, systemic congestion appears and RV failure manifests. In these cases, the console of the left-sided pVAD can present suction alarms. Normally, the pVAD works in auto mode after implantation, meaning that the device searches for the maximum achievable flow. When the RV is at risk of dysfunction, it would be better to set the pVAD in manual mode and increase the flow gradually to give the RV time to adapt to the increased venous return and flow conditions. RV failure can be detected with TTE searching for signs of systemic congestion (*Table 3* and *Figure 2*) and increased pressure in the right heart chambers which can manifest with RV dilation (that can be recognized by comparing RV size with that of LV) and inter-atrial and inter-ventricular septum bulging to the left.^50^ When RV failure is detected, apart from decreasing manually the left-sided pVAD flow, optimal ventilation setting and medical treatment (for instance with pulmonary vasodilators) to decrease RV afterload can be helpful.^50^ Moreover, an inotrope to increase RV contractility (for instance dobutamine) can avoid the need for a right-sided tMCS or other types of biventricular support.^21^

VA ECMO decreases systemic congestion and RV preload through the venous cannula and increases the CO and LV afterload through the arterial cannula.^51^ The failing LV may not be able to deal with the increased afterload and the pressure generated by the LV may not be able to open the aortic valve this would lead to LV distension with an increase in wall stress and decreased cardiac perfusion resulting in myocardial ischaemia. Moreover, LV distension can be accompanied by pulmonary oedema, blood stasis, and LV thrombus formation.^52^ TTE or TOE can help in monitoring these conditions and setup the right treatment to reduce LV afterload, tackle LV distension and maybe intensify anticoagulation therapy in case of LV thrombosis. LV afterload reduction can be achieved pharmacologically with systemic vasodilators and inotropes, by reducing VA-ECMO flow and, should these treatments fail, LV venting strategies may be used and include, for instance, atrial septostomy, IABP conterpulsation, and Impella placement. Finally, several echocardiographic parameters of cardiac function should be considered to decide when to start VA-ECMO weaning to make it successful. For instance, LVEF > 25% (or LVEF recovery), LV outflow tract velocity time integral ≥10 cm, lateral mitral annulus peak systolic velocity ≥ 6 cm/sec and also more advanced parameters, such as three-dimensional RV ejection fraction > 25% had been associated with successful VA-ECMO weaning.^53^

## Conclusions

Different tMCS can be used in emergency situations to support cardiac function or in elective cases to prevent the risks of acute heart failure. A rational approach based on invasive and non-invasive haemodynamic parameters and the use of vascular ultrasound for tMCS placement can reduce complications, optimize their management and allow taking full advantage of their potential benefits.

## Lead author biography


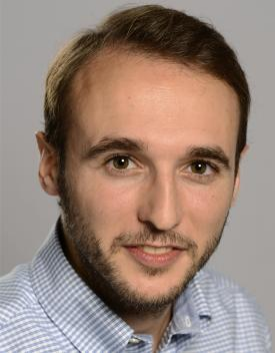
Dr. Federico Fortuni is a cardiologist specialized in cardiovascular imaging. He currently works as a consultant cardiologist at the Cardiology and Intensive Cardiac Care Unit of San Giovanni Battista Hospital in Foligno (Italy). He completed his cardiovascular medicine residency at the University of Pavia and San Matteo Hospital of Pavia. He completed a fellowship in advanced cardiovascular imaging at the Leiden University Medical Center (the Netherlands) and is currently completing a PhD programme in valvular heart disease and advanced cardiovascular imaging at the same university. He was awarded the Young Investigator Award in Clinical Cardiology by the European Society of Cardiology in 2020. He conducted several studies on multimodality cardiovascular imaging with more than 80 articles published in international peer-reviewed journals. He acts as a reviewer for several cardiology journals. He is a member of the Editorial Board of the ‘Journal of the American Society of Echocardiography’, ‘Frontiers in Cardiovascular Medicine’ and ‘Cardiovascular Ultrasound’. He is among the Associate Editors of the ‘European Heart Journal: Imaging Methods and Practice’.
